# Study on the Emulsifying Properties of Pomegranate Peel Pectin from Different Cultivation Areas

**DOI:** 10.3390/molecules24091819

**Published:** 2019-05-10

**Authors:** Hu Zhuang, Shang Chu, Ping Wang, Bin Zhou, Lingyu Han, Xiongwei Yu, Qinli Fu, Shugang Li

**Affiliations:** 1 Key Laboratory of Fermentation Engineering, Ministry of Education; Glyn O. Phillips Hydrophilic Colloid Research Center, Faculty of Light Industry; School of Food and Biological Engineering, Hubei University of Technology, Wuhan, 430068, Hubei Province, China; huzhuang9303@163.com (H.Z.); shang.chu@hbut.edu.cn (S.C.); zhoubin4111@163.com (B.Z.); hanlingyu1001@126.com (L.H.); 2 Production & Construction Group Key Laboratory of Special Agricultural Products Further Processing in Southern Xinjiang, College of Life Sciences, Tarim University; Alar 843300, China; wpaing513@163.com; 3 Wuhan Xudong Food Co., Ltd., Wuhan 430040, Hubei Province, China; yuxiongwei321@163.com (X.Y.); fuqinli111@sohu.com (Q.F.)

**Keywords:** pomegranate peel pectin, rheological behavior, emulsifying properties, interfacial adsorption

## Abstract

Pomegranate peel pectin is an important acidic anionic plant polysaccharide which can be used as a natural emulsifier. In order to study its emulsifying properties, this paper systematically analyses pomegranate peel pectin samples from Chinese Xinjiang, Sichuan and Yunnan provinces, through rheometer, interfacial rheometer, Zetasizer Nano-ZS and mastersizer. It is shown that pomegranate peel pectin can effectively reduce the oil-water interfacial tension, reaching an emulsion droplet size of only 0.507 μm, 0.669 μm and 0.569 μm, respectively, while the pectin concentration is 1.5% and the oil phase (MCT) is 10%. It has also shown that the extreme conditions of pH and ion strength can not significantly change its emulsion stability. However, freeze-thaw cycles can cause the pomegranate peel pectin emulsion to become less stable. Furthermore, the effects of decolourization, protein removal and dialysis on the emulsifying properties of pomegranate peel pectin are investigated using mastersizer rheometer and interfacial rheometer. It is found that the protein and pigment in pomegranate peel pectin have little effect on its emulsifying properties, while the results from dialyzed pectin show that the small molecule substances can reduce the emulsion particle size and increase the emulsion stability. The research outcomes of this study provide technical support for the further application of pomegranate peel pectin in the food industry.

## 1. Introduction

Pectin is a natural macromolecular polysaccharide substance widely present between plant cell walls [1], and it is often used as a thickener, gelling agent and emulsifier in the food processing industry. Essentially, it is a polysaccharide of linear structure, mainly composed of d-galacturonic acid, which are polymerized by α-1,4-glycosidic linkage to form a slightly acidic macromolecular polysaccharide, often with other neutral monosaccharide branches, such as d-galactose, d-sorbose, l-arabinose, l-rhamnose, xylose, etc. As an acidic anionic polysaccharide, pectin, was studied as early as 1986, when research done by Dea and Madden showed that sugar beet pectin had surface activity and could effectively prepare emulsion [2]. It was proven that pectin samples extracted from apple, pumpkin, sweet potato, hawthorn and watermelon have good emulsifying properties. However, the emulsifying properties of pomegranate peel pectin are relatively less studied [3,4,5,6,7]. Akhtar et al. analysed the depolymerized citrus pectin and found that pectin with molecular weight of 70 kg/mol has great emulsifying properties, and the pectin with that molecular weight can form a stable emulsion [8]. Leroux et al. found that citrus pectin can be used to effectively prepare emulsions, they also discovered that the impact factors for its emulsifying properties include the molecular weight, protein and acetyl contents [9]. Similar results have also been reported in the study of okra pectin, where the emulsifying properties of okra pectin is influenced by the protein content of the pectin emulsion [10]. Williams et al. obtained sugar beet pectin with a relatively high protein content by separating or depolymerizing crude sugar beet pectin. However, the sugar beet pectin obtained did not show better emulsifying properties, which indicated that the protein content is not necessarily the influencing factor of pectin emulsifying properties [11]. These studies showed that the structure and composition of pectin have great impacts on its emulsifying properties. However, these results are not applicable to pectins from all sources.

The pomegranate *(Punica granatum L.)* is a fruit-bearing deciduous shrub belonging to the Lythraceae family. It is known to be an excellent fruit that combines social benefits, economy, ecology, ornamental value and health benefits [12]. Pomegranate fruit is rich in nutrients such as carbohydrates, protein, amino acids, vitamins, pectin, ellagic acid, fatty acids and polyphenols [13]. Pomegranate fruit is mainly used in food industry for fresh foods, juice and wine production. Its processing byproduct is mainly pomegranate peel residue, which is rich in pectin content. Each 100 g of pomegranate peel residue contains 6~10 g of pectin, making it a good source of natural pectin. In recent years, many researchers around the globe have done a lot of research on the extraction and structure of pomegranate peel pectin [14,15,16] and a lot of basic results have been obtained on the separation and purification of it, as well as its biological activity. Yang et al. reported on the application of pomegranate peel pectin as an emulsifying agent [17]. However, there is still no systematic study on the differences in the emulsifying properties of pomegranate peel pectin from different cultivation areas and the possible impact factors for these properties. Therefore, this study used the edible sweet pomegranate cultivated from Xinjiang, Sichuan and Yunnan provinces in China as the experimental samples to analyse their emulsifying properties, as well as the impact factors for their emulsifying properties, by means of FTIR, rheology and interface research technologies. This study aims to use the analysis results to scientifically evaluate the intrinsic potential of pomegranate peel pectin as a natural emulsifier. The research outcomes can also provide theoretical basis and technical support for the application of pomegranate peel pectin in the food industry.

## 2. Results and Discussion

### 2.1. Composition and Structure Characterization of Pomegranate Peel Pectin

The composition and structure of pectin are key indicators in determining the functional properties of pectin. The composition and molar mass of pectin from different producing areas are shown in Table 1.

**Figure 1:**
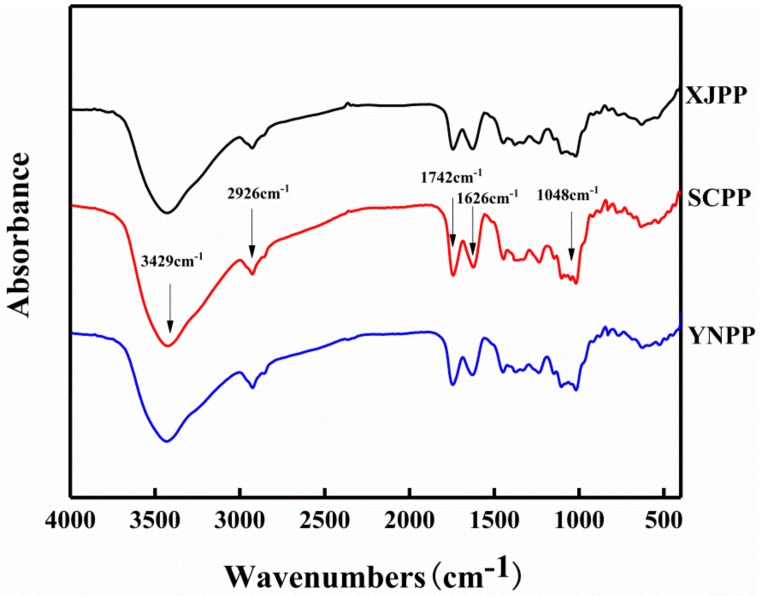
Pectin Fourier transform infrared (FTIR) spectrum.

As can be seen from Table 1, pomegranate peel pectin (PP) in Xinjiang, Yunnan and Sichuan is mainly composed of galacturonic acid (59.43~61.15%), glucose (20.52~26.81%), arabinose (6.57~10.79%) and galactose (4.13~8.02%); its molecular weight is 4.103 × 10^5^, 3.618 × 10^5^ and 4.384 × 10^5^ g/mol respectively; the protein content is between 2% and 3%; and the esterification degree is in the range of 52% to 58%. More than 50%, it is a high-methyl ester pectin, and its degree of acetylation is between 12% and 15%. It is a high-methyl ester pectin with a high degree of acetylation. According to J. Leroux et al., the degree of acetylation is one of the factors affecting the emulsifying properties of pectin [9]. It can be seen from Figure 1 that pomegranate peel pectin from different producing areas contains characteristic FTIR spectra of pectin, and there is little difference between them. Pappas et al. [18] reported that the DM of pectin is linearly correlated to the ratio of the peak area at 1742 cm^−1^ and the sum of the peak areas at 1742 cm^−1^ and 1626 cm^−1.^ It can be concluded that the order of pectin DM in the three regions is YNPP (58.74%) > SCPP (54.36%) > XJPP (52.27%), which highly matched the results from the chemical determination method.

### 2.2. Analysis of Emulsion Properties

#### 2.2.1. Analysis of Emulsification and Storage Stability of Pectin

**Figure 2:**
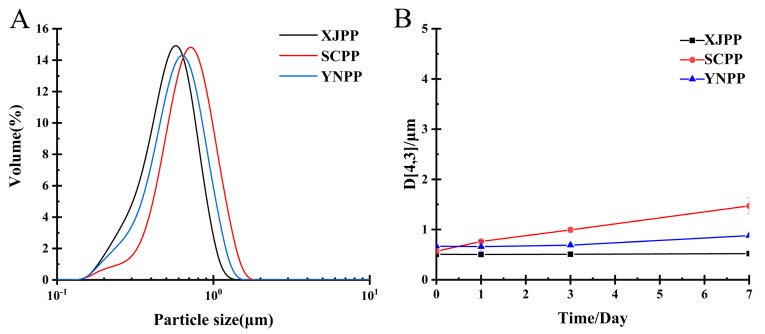
Particle size distribution of 1.5% pomegranate peel pectin emulsion (**A**), droplet size change during seven days of storage under 60 °C (**B**) (The results in Figure 2B were from samples prepared at a later time.).

The oil-in-water emulsion was prepared using PP (concentration: 1.5%) as an emulsifier and medium-chain triglycerides (MCT) (10%) as the oil phase. The particle size distribution and the seven-day storage change of the emulsion were as shown in Figure 2.

It can be seen from Figure 2A that the three pectin emulsions have a small particle size and their distributions all have a unimodal bell shape, indicating that they have good emulsifying properties. There is only a slight change observed in the particle size distributions of pectin emulsions. Storage at 60 °C for seven days, through the D[4,3] change of the emulsion particles (Figure 2B), PP emulsion stability is good and only a small change in YNPP D[4,3] was observed during the seven-day accelerated storage period. However, there was no creaming phenomenon. Among them, XJPP and SCPP emulsions did not change in the size of emulsion D[4,3] during the accelerated storage period of 1~3 days, which was extremely stable. Compared with the beet pectin reported by Hai-ming Chen et al., although it has good emulsifying properties, there are obvious defects in poor emulsion stability [19]. Therefore, PP has obvious advantages in both emulsification and emulsion stability.

#### 2.2.2. Rheology Analysis

**Figure 3:**
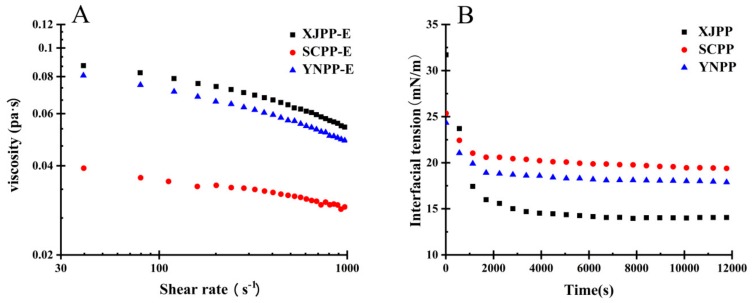
Pectin emulsion viscosity changes with shear rate (**A**), pectin adsorption curve at oil-water interface (**B**) (25 °C).

Rheological and interfacial rheological properties are important means to characterize the emulsion emulsification and emulsion stability. The rheological properties of PP are shown in Figure 3.

As shown in Figure 3A, the apparent viscosity of the emulsion showed a decreasing trend with the increase of the shear rate. When the pectin concentration was 1.5% and the shear rate was 40 1/S, the order of viscosity of PP-E from different areas was XJPP-E (0.087 pa·s) > YNPP-E (0.081 pa·s) > SCPP-E (0.039 pa·s), which is consistent with the order of emulsion stability data, indicating that when the viscosity of PP-E is higher, the emulsion is more stable. This finding is consistent with the polysaccharide emulsion results reported by Arash Koocheki, Dickinson, Gart et al. To be more exact, within a certain concentration range, the increase of viscosity will cause the oil droplet movement rate to decrease, so that the emulsifier has sufficient time to be adsorbed at the oil-water interface to stabilize the emulsion system [20,21]. Meanwhile, the viscosity of the aqueous phase was increased by the emulsifiers that are not adsorbed on the interface, inhibiting the movement of the droplets in the emulsion, eventually resulting in reduced flocculation, aggregation and delamination of the particles in the emulsion. The stability of emulsion is a result of two factors: Enough emulsifier absorbed at the oil-water interface, and the excess emulsifier increases the viscosity.

As shown in Figure 3B, at 25 °C, the interfacial tension of all samples decreased with time and finally reached a plateau value, which suggested that pectin molecules were gradually adsorbed at the oil/water interface to reduce interfacial tension. The results of dynamic interfacial tension were measured for the pomegranate peel pectins from different areas. The equilibrium interfacial tension is (XJPP 14.05 mN/m; SCPP 19.39 mN/m; YNPP 17.88 mN/m), which may be related to the structure and composition of pectin. The presence of hydrophobic groups (methyl and acetyl) and the presence of protein residues can act as hydrophobic anchors and promote pectin adsorption at the interface resulted in a reduction in interfacial tension [22,23,24].

#### 2.2.3. Influence of Environmental Conditions on Stability of Pomegranate Peel Pectin Emulsion

**Figure 4:**
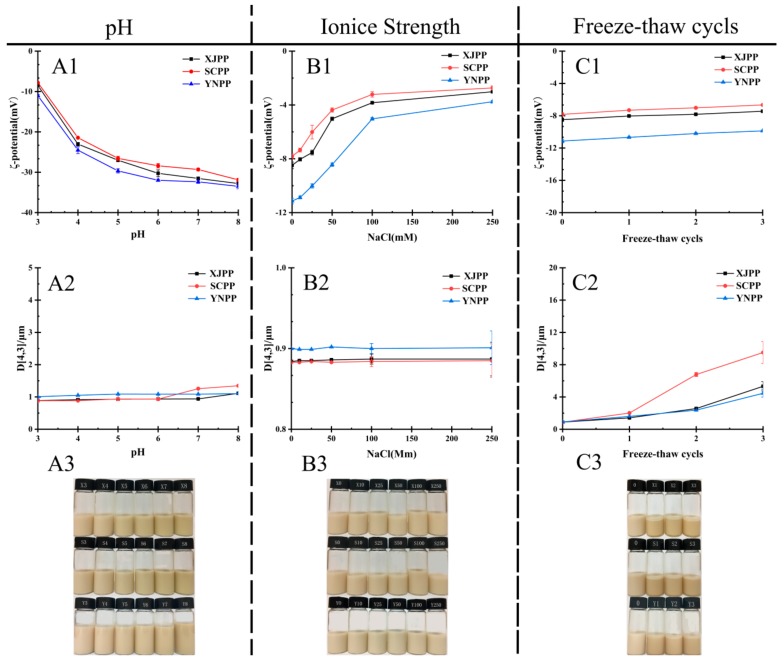
Influence of pH on emulsion ξ-potential (**A1**), average particle size (**A2**), emulsion stability (**A3**), Na+ on emulsion ξ-potential (**B1**), average particle size (**B2**), emulsion stability effect (**B3**), freeze-thaw cycles on emulsion ξ-potential (**C1**), average particle size (**C2**) and emulsion stability (**C3**). The emulsion was observed after seven days of storage at room temperature.

In the food industry production, the emulsion is often a multi-phase, multi-structure complex system. Its stability is often affected by many factors such as pH, ionic strength and freeze-thaw cycles. The experiment explored the pH of 3~8, respectively. The effect of Na+ ion and three freeze-thaw cycles on the stability of pomegranate peel pectin emulsion from different producing areas is shown in Figure 4. 

It can be seen from Figure 4A1,A2 that as the pH increases, the potential of the PP emulsion shows a significant downward trend, while the particle size hardly changes. This may be due to the presence of methyl esterification of galacturonic acid in pectin. There is acetylation at the C2 and C3 positions. Therefore, the acid ions are less, and the steric hindrance and viscosity characteristics play a favourable role in maintaining emulsion stability. On the contrary, the change of electrostatic repulsion has little effect on the change of emulsified particles. Arabic gum also had similar properties, when the pH value changes within the range of 3 to 6, the particle size of the emulsion does not change significantly [25]. Through the results of its storage stability test, it can be seen that after seven days of storage at room temperature (Figure 4A3), the PP emulsion still shows strong stability, and no creaming phenomenon occurs.

It is also shown in Figure 4B1,B2 that as the concentration of Na+ increases, the absolute value of the ξ-potential of the PP emulsion decreases [26], while the particle size does not change much, and the PP emulsion has no obvious cream after storage for seven days at room temperature. Analysis of the phenomenon (Figure 4B3), which may be due to the electrostatic shielding effect of Na+ ions, which reduces the amount of charge on the surface of the emulsion droplets; and the steric hindrance of PP self-composite layer and higher viscosity and hydrophilic hydration ability, so that it has enough protective barrier without aggregation.

As presented in Figure 4C1,C2 that after three freeze-thaw cycles, the average magnitude of charge decreased with repeated freeze-thaw cycles. There is an obvious change observed in the particle size results. After seven days of storage at room temperature, it was found that the emulsion of PP showed a slight creaming phenomenon (Figure 4C3). The cause of the potential change may be caused by a change in particle aggregation after the freeze-thaw cycle.

From the above, it is proven that pomegranate peel pectin emulsion is not sensitive to environmental conditions such as pH change and Na+ concentration, and it can maintain its good emulsion stability under these circumstances. However, its stability can be affected by freeze-thaw cycles.

### 2.3. Analysis of Emulsification of Pomegranate Peel Pectin

#### 2.3.1. Influence of Purification Treatment on Emulsification and Storage Stability of PP Emulsion

**Figure 5:**
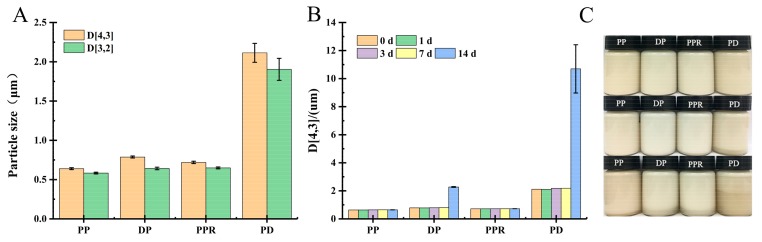
Different particle size of pomegranate peel pectin PP fresh emulsion (**A**), 14 days of storage under 60 °C (**B**), and the appearance of emulsion in storage environment (**C**: 60 °C storage from top to bottom zero days, seven days, 14 days).

In order to explore the emulsification and emulsion stability of PP emulsion, in the emulsion system with PP concentration of 1.0% and oil phase MCT of 10% (*v*/*v*), the experiment focused on decolorization, protein removal and dialysis treatment on PP emulsion. The effects of emulsifying properties and emulsion stability are shown in Figure 5.

It is shown in Figure 5 that decolorization and protein removal have little effect on the particle size of PP emulsion, and dialysis makes the particle size of PP emulsion increase significantly. The change of storage stability is that protein removal treatment has less influence on it, and decolorization and dialysis. The treatment has a great influence on it, and there is a more obvious phenomenon of cream precipitation.

#### 2.3.2. Influence of Purification Treatment on the Viscosity of PP Emulsion

**Figure 6:**
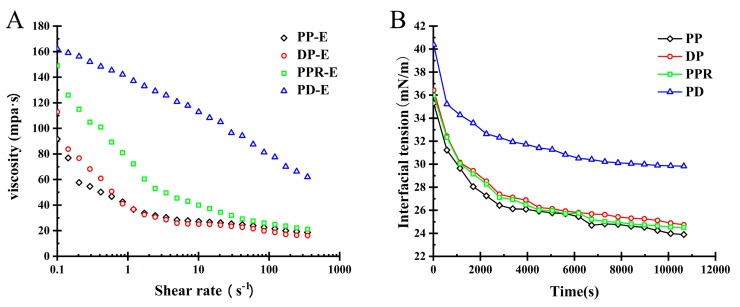
Viscosity of PP emulsion with different purification treatments as a function of shear rate (**A**), adsorption curve of PP at oil-water interface for different purification treatments (**B**) (25 °C).

The effects of decolorization, protein removal and dialysis on the emulsification and emulsion stability of PP emulsion were investigated. The results are shown in Figure 6A.

After each purification treatment, the viscosity of the sample decreases with the increase of shear rate, and its viscosity is greater than PP-E. Especially after dialysis, the viscosity of PP-E increases most obviously. This may be because pomegranate peel pectin is a hydrophilic polymer. The compound is purified to increase the concentration of the emulsion, and the gap between the molecules causes molecular aggregation and entanglement due to the hydrophilic action, thereby greatly increasing the viscosity of the emulsion.

#### 2.3.3. Influence of Purification Treatment on Rheological Behaviour of PP Interface

The interfacial activity directly affects the emulsifying ability of the polymer surfactant. The oil-water interfacial tension with time at 25 °C for PP samples after different purification treatments were presented in Figure 6B.

It was shown by the interface adsorption line that the purification treatment, such as decolorization, protein removal and dialysis, all caused a decreasing trend in the interfacial tension as the adsorption time increased. The initial interfacial tension of pomegranate peel pectin after decolorization and protein removal decreased from 35.35~36.42 mN/m to 23.89~24.74 mN/m, while the initial interfacial tension of PP before dialysis treatment was 40.38 mN/m and decreased to 29.83. mN/m after it. The results demonstrated that the decreasing rate of interfacial tension at the oil-water interface induced by PP was relatively slower and less reduced after the dialysis treatment removed the small molecular substance.

#### 2.3.4. Analysis of Interface Adsorption Components

**Figure 7:**
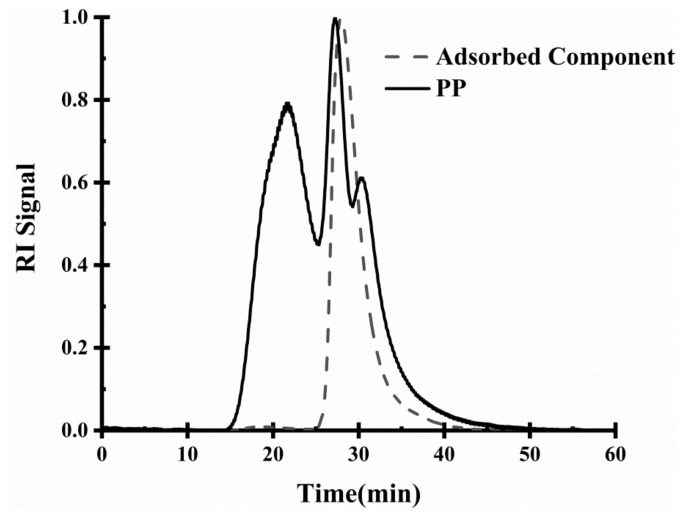
Gel permeation chromatography (GPC) elution profile of initial PP and PP component adsorbed at the emulsion interface (parallax signal).

The adsorption component of PP on the MCT interface was analysed by GPC-MAllS. The initial PP and the gel permeation chromatography (GPC) elution profile of the PP component adsorbed at the emulsion interface are shown in Figure 7.

As presented in Figure 7, the peaking time of the components of the purified PP without decolorization, protein removal and dialysis are between 15 and 48 min, and the peaking time of the adsorbing component is only between 25 and 42 min. Since GPC-MALLS sequentially elutes the polymer in the solution according to the molecular hydrodynamic volume order [27,28], it is shown that the PP small molecule substance is the main adsorption component at the emulsion interface. As the results suggested, dialysis treatment could have a negative impact on the emulsifying properties of PP.

## 3. Materials and Methods

### 3.1. Materials

Fresh sweet pomegranate fruit samples were cultivated from Hetian City, Xinjiang Uygur Autonomous Region, Mengzi Autonomous Prefecture, Yunnan Province, and Liangshan Autonomous Region, Sichuan Province, China. The three different pomegranate peels were collected from the fruits and grinded after oven-drying.

### 3.2. Preparation of Pomegranate Peel Pectin

#### 3.2.1. Extraction of Pomegranate Peel Pectin

The extraction of pomegranate peel pectin was carried out according to the method of Pereira et al. [15] with some modifications. Different from the original method, the extraction parameters were adjusted to the following: solid-liquid ratio 1:20g/mL, pH value 2.0 (adjusted using 1M HCL), extraction temperature: 80 °C, extraction time: 120 min. Three kinds of pectin samples were obtained after the extraction steps: Xin jiang pomegranate peel pectin (XJPP), Si chuan pomegranate peel pectin (SCPP), Yun nan pomegranate peel pectin (YNPP).

#### 3.2.2. Purification of Pomegranate Peel Pectin

The crude PP extract was obtained according to the method mentioned in 3.2.1, and then the pigment in the crude pectin was removed by D101 macroporous adsorption resin to obtain decolorized pectin (DP). The D101 resin decolorizing method was adopted from the literature of Shu [29]. 5% (*w*/*v*) of D101 resin was added into the PP sample, which was then placed in a thermo shaker at 40 °C for 4 h with a rotation speed of 150 rpm/min. In order to obtain the decolorization rate, the pectin samples before and after decoloring treatment were diluted by 200 times respectively, then they were scanned by UV spectrometer to obtain their spectrum. The decolorization rate was obtained by the comparison of the absorption peaks at 380 nm. The decolarization rate was about 71% for this study. For some other crude PP samples, the protein was removed using Sevage method to obtain PP after protein removal (PPR). One fourth volume of sevag reagent (1-butanol/chloroform, *v*/*v* = 1/5) was added to the sample solution. The mixture was shaken vigorously and then centrifuged. The aqueous phase was recovered. The sSvag assay was repeated until there was not precipitate. The protein removal was verified by the Coomassie Brilliant Blue G-250 method. The protein removal steps were repeated until no protein could be detected. Also, some other crude PP had the small molecular impurities removed by dialysis technology using (MW: 7000~14,000) dialysis bag to obtain PP after dialysis treatment (PD). The PP sample was placed into a pre-treated dialysis bag, and then was dialyzed in distilled water for 48 h. The water was replaced by fresh distilled water every 2 h to ensure there was sufficient osmotic pressure for the small molecular substances to move out.

### 3.3. Determination of the Degree of Acetylation of Pectin Esterification

The degree of methyl esterification of PP was determined using the method mentioned in the previous research of Virk [30] and Pinheiro [31] with some modifications. 0.1 g of pectin was measured, mixed with 1 mL of ethanol, and then 1 g of NaCl was added into the mixture, which was dissolved with ultrapure water to make a 100 mL solution. After the mixture was fully dissolved, phenolphthalein was used as an indicator, the solution was titrated with 0.1 mol/L NaOH until the colour turned pink (pH 7.5), and no discoloration was observed for 30 s. The volume of NaOH used was recorded as V1. 15 mL of 0.25 mol/L NaOH was added to the solution and stirred at room temperature for 30 min. 15 mL of 0.25 mol/L hydrochloric acid solution was added, and then titrated with 0.1 mol/L NaOH until the colour turned pink, and no discoloration was observed for 30 s. The volume of NaOH used was recorded as V2. The formula used to calculate the degree of methyl esterification (DM) was the following:

DM (%) = 100 × V2/V1 + V2,(1)

The degree of acetylation of PP was determined by the method of Virk et al. [15], with some modifications. 0.1 g of pectin was mixed with 20 mL of 0.125mol/L NaOH until the pectin was completely dissolved, then the mixture was dissolved with ultrapure water to make a 50 mL solution. Twenty mL of the solution was poured into a distillation flask, and 20 mL of magnesium sulfate-sulfuric acid solution (180 mL of saturated magnesium sulfate solution contained 1.5 g of sulfuric acid) was added. The solution was fully mixed and then concentrated by rotary evaporation at 40 °C. The distillate was collected and titrated with 0.05 mol/L NaOH, with phenolphthalein being used as the indicator. Titration continued until the colour turned pink and no discoloration was observed for 30 s. The volume of NaOH was recorded as V. A magnesium sulfate-sulfuric acid solution distillate, without any sample, was used as a blank. The determination of V1 and V2 was done following the same steps described in the above method for DM. The formula used to calculate the degree of acetylation (DA) was the following:

DA (%) = 100 × 0.05 × V/V1 + V2,(2)

### 3.4. FTIR Analysis

The dried pectin was mixed with potassium bromide (KBr) at a ratio of 1:250 (*w/w*), and then pressed into a translucent pan for Fourier transform infrared spectroscopy (FTIR). Spectra were obtained from a potassium bromide sample pan in a range of 4000 to 400 cm^−1^ using a Perkin Elmer Spectrum RXI FTIR spectrometer (Perkin Elmer Instruments, Waltham, MA, USA) with a resolution of 4 cm^−1^.

### 3.5. GPC-MALLS Characterization

The method of Shengping Xiang [32] was used to characterize the molecular weight distribution of PP from different origins. GPC-MALLS was used to characterize the PP from different areas as well as the PP adsorbed onto the emulsion interface and that present in aqueous phase. The system consists of a Superose 6 10/300GL column (GE Healthcare Co., Boston, MA, USA) and a DAWN HELEOS multi-angle light scattering detector (Wyatt Technology Corporation, Santa Barbara, CA, USA), an SPD-10Avp series UV detector (Shimadzu Technologies, Kyoto, Japan) operated at 214 nm, an Optilab rEX refractometer (Wyatt Technology Corporation, Santa Barbara, CA, USA) at 25 ± 0.2 °C. 0.2 M NaCl aqueous solution containing 0.03% NaN3 with a flow rate of 0.4 mL/min was used as an eluent after filtration through a 0.45 µm Millipore filter. A dn/dc value of 0.142 mL/g was taken for PP.

### 3.6. Determination of the Composition of Pomegranate Peel Pectin Monosaccharides

Hydrolysis of the pectin samples: Pectin samples were dissolved in deionized water at the concentration of 2 mg/mL, 1 mL pectinase was added, and the samples were incubated in a 40 °C water bath for 24 h, and then 1 mL of 6M H2SO4 solution was added and the samples were placed in a boiling water bath for 2 h. After cooling, the pH was adjusted to neutral with NaOH. After diluting to a certain extent, the samples were passed through a 0.22 μm water film and prepared for analysis.

The monosaccharide composition of pectin polysaccharide was investigated by HPAEC-PAD method to characterize its monosaccharide composition. The settings and conditions were as the following: separation column was Carbo PacPA-1 (inner diameter 4 mm, column length 250 mm), mobile phases were A: 250 mM NaOH; B: 1 M NaAc; C: Deionized water. The flow rate was 1 mL/min, the column temperature was 30 °C, and the injection volume was 25 μL. In the integrated pulse amperometric detector, Au was the working electrode and Ag/ACl was the reference electrode.

The gradient procedure for neutral monosaccharide and uronic acid elution is as follows [25]: 5 mM NaOH (0-20 min), 5~100 mM NaOH (20~30 min), 100 mM NaOH + 0~100 mM CH3COONa (30~50 min, 200 mM NaOH (50~60 min).

The calculation of the mole percentage of the HG and RG-I regions of pectin is based on the calculation method of Sakni et al. [33]. The formula used is as follows:

HG (mol%) = GalA (mol%) − Rha (mol%),(3)

RG-I (mol%) = [GalA (mol%) − HG (mol%)] + Rha (mol%) + Gal (mol%) + Ara (mol%),(4)

### 3.7. Emulsion Properties

#### 3.7.1. Preparation of Emulsion

In in ultrapure water in 0.02% sodium azide, 1.5% (*w/w*) pectin was dissolved and stirred at 25 °C for 16 h. Twenty-seven mL of 1.5% pectin solution and 3 mL of medium chain triglyceride oil (MCT) were processed by a high-speed shear (IKARO10 Digital Ultra-Turraxhomogensier, IKA Werke GmbH & Co., Staufen, Germany) at 20,000 rpm for 2 min. The mixed solution was homogenized 3 times by high pressure micro-jet homogenizer (M-110L, MFIC Co., Boston, MA, USA) under 75 Mpa.

#### 3.7.2. Emulsion Droplet Size

The particle size distribution of the emulsion was measured using a Malvern mastersizer 2000 instrument (Malvern Instruments, Malvern, UK), measured by wet method, universal mode, and the pump speed was set to 2000 r/min. The measurement results can be analysed to obtain the volume distribution of the emulsion droplets, the surface area weighted average diameter D[3,2] and the volume weighted average diameter D[4,3]. The dispersed phase is MCT and the continuous phase is water. Each sample was measured in parallel three times and finally averaged. The emulsion particle size distribution of the emulsion storage for zero, one, three and seven days was measured under the environment of 60 °C.

#### 3.7.3. Determination of the Zeta Potential of the Emulsion

The zeta potential value of the composite solution prepared above was measured using a Malvern Nano Zetasizer instrument (Malvern Instruments, Malvern, UK) equipped with a 4 mW He/Ne laser generator, which was launched at 633 nm. When the instrument determines the zeta potential, an electric field is applied between the two ends of the solution.

ξ = 3ηUE/2εf(ka),(5)

where: η is the solution viscosity, ε is the dielectric constant of the solvent, and f (ka) is the Henry function. The ξ-potential indicating the electrophoretic mobility was detected by a Zetasizer Nano-ZS type nanoparticle size and potential analyser. Prior to the measurement, the emulsion was diluted 1000 times with ultrapure water to avoid multiple scattering, and the measurement temperature was 25.0 ± 0.2 °C. Each sample was measured in parallel three times and finally averaged.

#### 3.7.4. Analysis of Emulsion Rheology

The emulsion viscosity was measured by a Haake Rheostress 6000 rheometer (Thermo Fisher Scientific, Waltham, MA, USA) in the experiment. The entire experiment used a geometric lamina titanium rotor with a cone diameter of 60 mm (model: C60Til, cone angle 1o, measuring spacing 0.052 mm). The emulsion viscosity of pomegranate peel pectin prepared at different concentrations of 1.5% was determined by steady-state shear test, and the experimental temperature was set to 25.0 ± 0.2 °C by circulating water bath. Experimental parameters: Shear rate 0.01~1000 s^−1^, set logarithmic mode to collect data.

#### 3.7.5. Interface Properties

In this paper, the Tracker-type bubble/droplet profile analyser (TRACKER, TECLIS Co., Lyon, France) was used to measure the interfacial tension of XJPP, YNPP, SCPP and CP after adsorption at the MCT-water interface with temperature at 25 °C. The analyser measures the change of the interfacial tension with the adsorption time, in order to collect details about the adsorption process of the active surface material at the interface.

A 5.0 mL pectin dispersion (0.01% *w/w*) was weighed into the sample cell at 25 °C, the sample needle was immersed in the aqueous phase and further 10 μL of oil droplets were formed by motor control. The test frequency is 0.05 Hz, and the amplitude is 10%. The whole measurement time lasted for 3 h, and the pomegranate peel pectin was fully adsorbed at the interface after 3 h. During the inspection process, the entire inspection system should be balanced to avoid external vibration interference measurement.

Analysis of interface adsorption components: according to the method of Nakauma et al. [34], the components adsorbed on the oil-water interface of the PP emulsion were replaced by the surfactant SDS (sodium dodecyl sulphate), and they were transferred to the aqueous phase. The lower aqueous phase was recovered and its molecular weight characteristics were determined by GPC-MALLS.

### 3.8. Analysis of Emulsion Stability in Extreme Environments

#### 3.8.1. Influence of pH Values on Emulsion Stability

The PP emulsions (each 10 mL) had their pH adjusted, in order to obtain PP emulsions of different pH (3.0, 4.0, 5.0, 6.0, 7.0, 8.0) using 0.1 M HCl/NaOH. The emulsion particle size and zeta potential were characterized by Mastersizer and Zetasizer after standing in room temperature for 24 h.

#### 3.8.2. Influence of Ionic Strength on Emulsion Stability

The PP emulsions (each 10 mL) was diluted with sodium chloride solution (1 mol/L) to obtain different sodium chloride concentrations (0, 10, 25, 50, 100, 250 mmol/L) within the emulsions. The emulsion particle size and zeta potential were characterized after standing in room temperature for 24 h.

#### 3.8.3. Influence of Freeze-Thaw Cycle on Emulsion Stability

The PP emulsions (each 10 mL) were transferred into the bottles and incubated in a −22 °C freezer for 22 h. Then, the emulsions were thawed in a 40 °C water bath for 2 h. This freeze-thaw cycle was repeated three times. The emulsions were characterized after these three cycles.

## 4. Conclusions

The study found that the pomegranate peel pectin emulsions in Xinjiang, Sichuan and Yunnan have smaller particle sizes. The results showed that the pectin reduced the oil-water interfacial tension to a certain extent. Furthermore, the pectin emulsions showed excellent stability under extreme environments such as pH, Na^+^ concentration, indicating that pomegranate peel pectin from different cultivation areas performed well as emulsifier and maintained good emulsion stability. However, it was observed that the stability of the pectin emulsions was affected by freeze-thaw cycles. In addition, it can be seen from the results that decolorization and protein removal have little effect on the emulsifying property, while the dialyzed small molecular substance has an enhancing effect on both the emulsifying property and the emulsion stability. The experimental results effectively confirmed the market application prospect of pomegranate peel pectin as a natural emulsifier. The outcomes of this study can also provide great technical support for the further development and utilization of pomegranate peel pectin, and for the future research on its emulsifying mechanism.

## Figures and Tables

**Table 1:** Composition and molecular weight of pectin monosaccharides from pomegranate peel from different areas.

	XJPP	SCPP	YNPP
Monosaccharide composition (%)			
Rha	1.88 ± 0.06	1.24 ± 0.11	1.35 ± 0.09
Ara	9.16 ± 0.15	10.79 ± 0.22	6.57 ± 0.14
Gal	7.78 ± 0.07	8.02 ± 0.19	4.13 ± 0.16
Glu	21.08 ± 0.11	20.52 ± 0.43	26.81 ± 0.23
Gala	60.11 ± 0.25	59.43 ± 0.76	61.15 ± 0.38
HG	58.23	58.19	59.80
RG-I	20.70	21.29	13.40
DA(%)	14.58 ± 1.37	13.21 ± 0.74	12.24 ± 1.21
DM(%)	52.27 ± 1.07	54.36 ± 0.82	58.74 ± 0.27
Protein(%)	2.41 ± 0.21	2.01 ± 0.13	2.82 ± 0.38
Mw (g/mol)	4.103 × 10^5^	3.618 × 10^5^	4.384 × 10^5^
